# A simple and rapid diagnostic method for 13 types of high-risk human papillomavirus (HR-HPV) detection using CRISPR-Cas12a technology

**DOI:** 10.1038/s41598-021-92329-2

**Published:** 2021-06-17

**Authors:** Jiaojiao Gong, Guanghui Zhang, Wangguo Wang, Liping Liang, Qianyun Li, Menghao Liu, Liang Xue, Guanghui Tang

**Affiliations:** 1 Yaneng Biotech, Co., Ltd., Fosun Pharma, Shenzhen, China; 2 Clinical Laboratory, Shenzhen Hengsheng Hospital, Shenzhen, China; 3 Department of Infectious Diseases, The Second People’s Hospital of Shangrao, Shangrao, Jiangxi Province China; 4 Department of Neurology, Hwa Mei Hospital, University of Chinese Academy of Sciences, Ningbo, China; 5 Nanobiological Medicine Center, Key Lab of Fuel Cell Technology of Guangdong Province, School of Chemistry and Chemical Engineering, South China University of Technology, Guangzhou, China

**Keywords:** Biological techniques, Biotechnology, Microbiology

## Abstract

Cervical carcinoma is the second most common cancer in women worldwide with greater than 99% of the cases caused by human papillomaviruses (HPVs). Early detection of HPVs especially the high risk types (HR-HPVs) are essential to prevent the disease progression. The existing methods for HPV detection, such as qPCR are of high sensitivity and specificity, but the need for expensive machinery and well-trained personnel slow down the disease detection. The emerging Cas12a-based method presents a new technique for nucleic acid detection. However, it is time-consuming and labor-intensive when used for HPV detection, as several reactions are required in order to identify multiple HPV infections. We herein present a non-genotyping method for 13 types of HR-HPV detection in a single reaction by combining the isothermal recombinase polymerase amplification (RPA) method with CRISPR-Cas12a technology. The result could be achieved in 35 min with high sensitivity (500 copies per reaction). This assay represents great advances for the application of RPA-Cas12a system and holds a great potential to address the key challenges facing the HPV diagnostics.

## Introduction

Human papillomavirus (HPV) infection is a major causative agent of cervical cancer in women^1^. In 2018, approximately 311,000 women died from cervical cancer and more than 85% of these deaths occurred in low- and middle-income countries^2^. HPV types can be classified into high risk (HR) and low risk (LR). The HR types (e.g., types 16, 18, 31, 33, 35, 39, 45, 51, 52, 56, 58, 59, 68) are known to be closely associated with pre-neoplastic lesions and carcinomas, while the LR types (e.g., types 6, 11, 40, 42, 43, 44, 54, 61, 70, 72, 81) tend to cause warts^3^. Early detection of the HR-HPVs using a simple and rapid method is extremely important for therapeutic action against cervical cancer particularly in areas where specialized equipment is not available^4^.

To date, a variety of primer combinations amplifying different regions of the HPV genome have been developed using quantitative polymerase chain reaction (qPCR) technology. The MY09/MY11 primer set and the GP5+/GP6+ primer set, both targeting the conserved L1 region, are the most frequently used amplification systems for the detection of HPV DNA in clinical samples^5,6^. The former of which includes several degenerate nucleotides in each primer while the latter consists of a fixed nucleotide sequence for each primer, and both of them have shown capability of amplifying a wide spectrum of HPV types^7^.

The qPCR technology, although known as an effective tool for detection and typing of viruses, is faced with disadvantages such as complicated procedures and requirement of expensive machinery^8,9^. The emerging isothermal amplification technologies, recombinase polymerase amplification (RPA) and loop-mediated isothermal amplification (LAMP) for examples, have been employed to diagnose HPVs^3,10^. These methods do not rely on thermal cycling and therefore are suitable for resource-restricted areas. However, they are time-consuming and labor-intensive when genotyping specimens containing multiple HPV types, as only one type of HPV could be detected in a single reaction. In some cases, it might not be necessary to genotype all individual HPV types, but rather detecting all HR-HPVs as a whole.

The clustered regularly interspaced short palindromic repeats (CRISPR) and Cas (CRISPR associated proteins) are adaptive immune systems in archaea and bacteria^11,12^. Some Cas nucleases display strong collateral activities after binding to their specific *cis* targets, which has been fully evaluated for diagnostic use^11,13^. In combination with RPA amplification, CRISPR-Cas12a system has been shown to permit a single molecule detection^14,15^.

**Figure 1:**
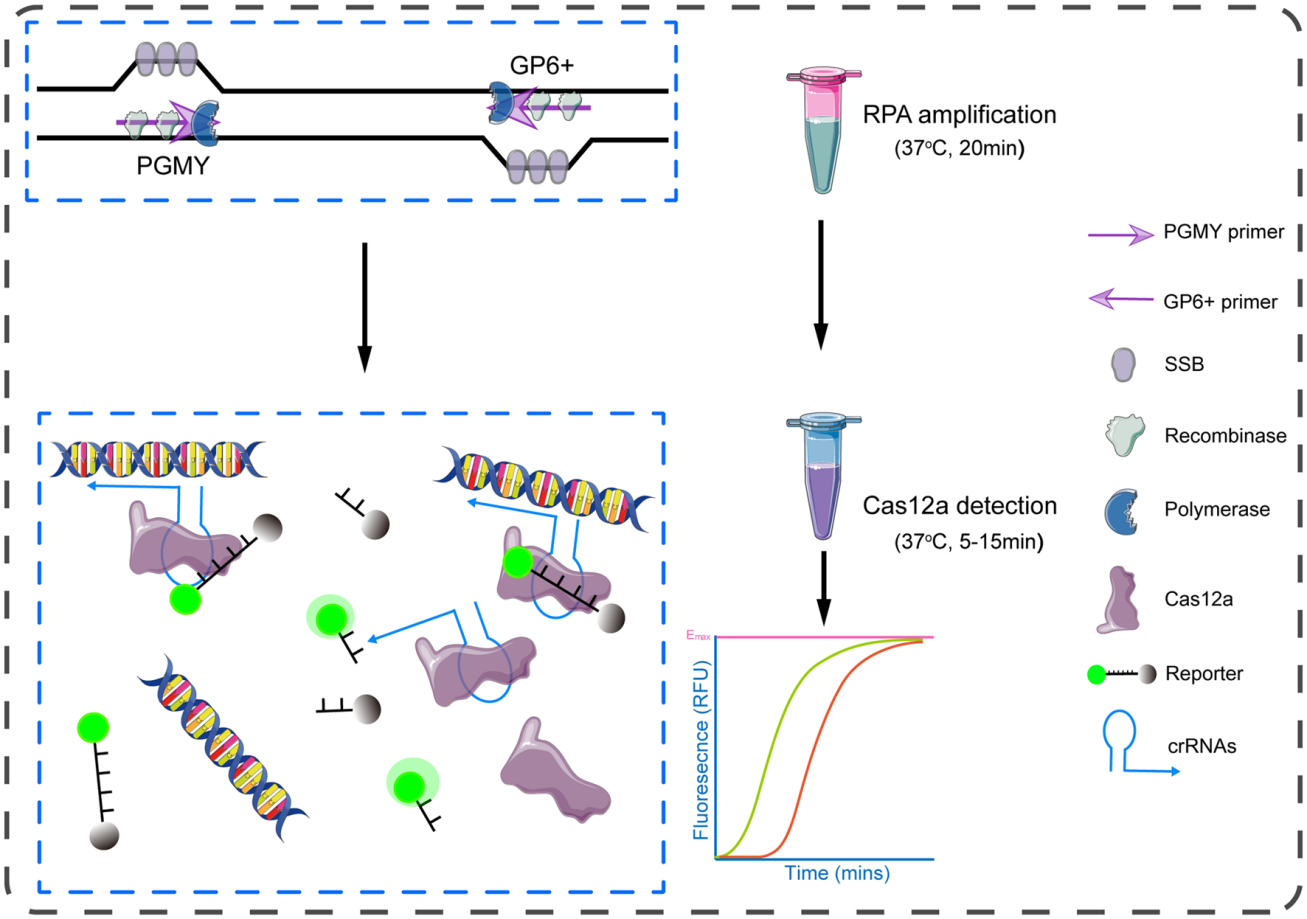
Illustration of the RPA-Cas12a method for HR-HPVs detection. The system includes an RPA amplification with a pool derived from PGMY/GP6+ primer set that is able to amplify 13 types of HR-HPV in a reaction, and a Cas12a detection with a crRNA pool. The results can be achieved within 35 min using a fluorescent reader.

In current study, we present a non-genotyping approach for 13 types of HR-HPVs detection using RPA-Cas12a system, with which the infection status of an individual by HR-HPVs could be clearly identified. Our assay consists of an RPA amplification using the primer pool derived from PGMY/GP6+ primer set, and a detection mediated by CRISPR-Cas12a (Fig. 1). To our knowledge, this is the first report realizing multiple HPV testing in a single reaction with the RPA-Cas system and hence represents significant advances for the application of this system. Because the present assay is fast and needs minimal equipment, its application may help the triage procedure of the cervical cancer screening.

## Results and discussion

**Figure 2:**
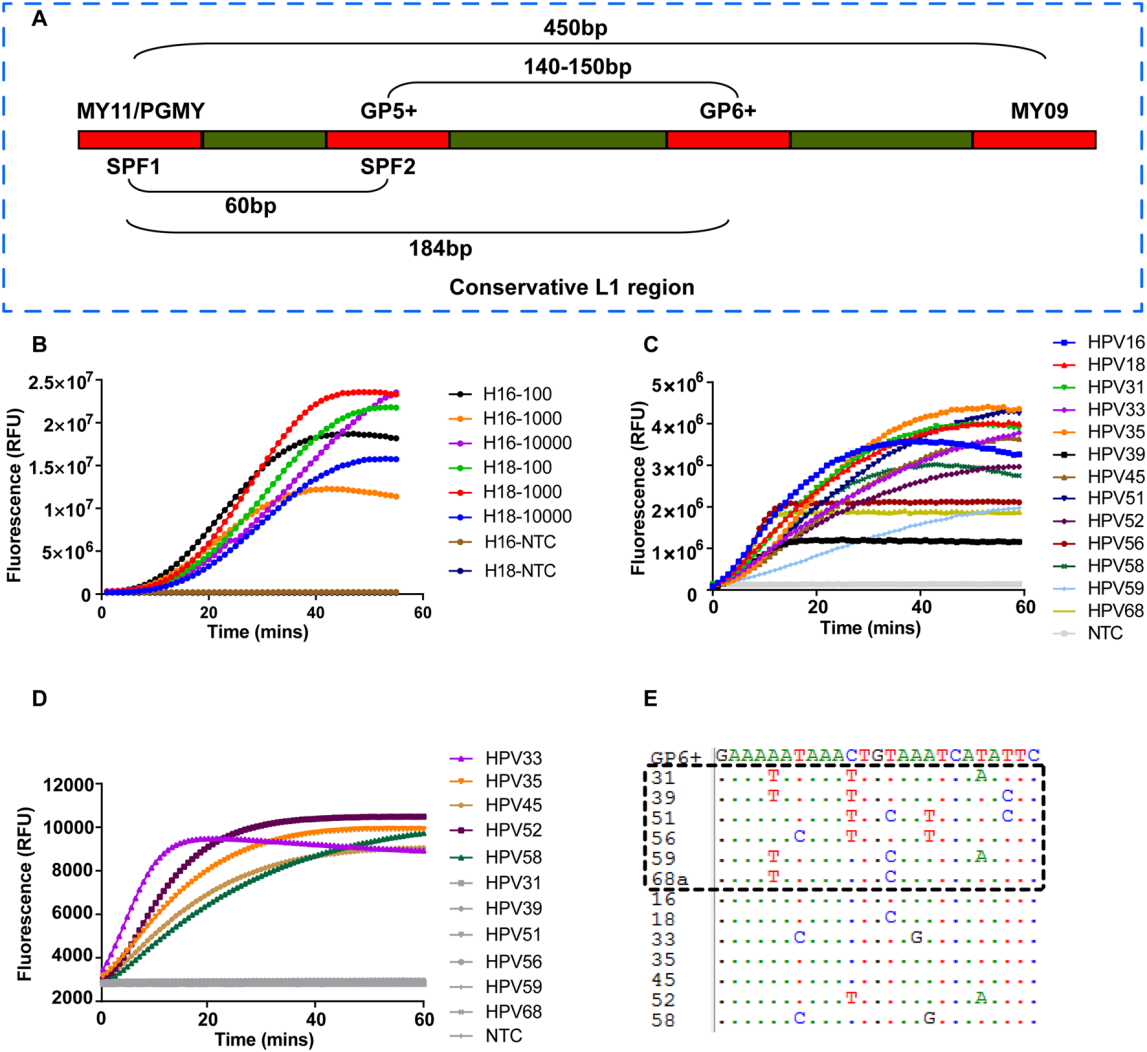
Evaluation of the PGMY/GP6+ primer pool. (**A**) Schematic showing the relative position of the primer sets locating in the conservative L1 region of HPV. (**B**) Plasmids with different copy numbers of HPV16 and HPV18 were amplified by RPA reaction using the PGMY/GP6+ primer pool. *NTC* no-template control. (**C**) Real-time fluorescence detection of HPVs with the crRNA pool containing 11 crRNAs. For a single reaction, 5 μg of each individual HPV plasmid was used. *NTC* no-template control. (**D**) The PGMY/GP6+ pool was tested using 10,000 copies of the indicated HPV plasmids for each RPA reaction. The RPA reaction was performed at 37 °C for 20 min and followed by Cas12a detection at 37 °C for up to 60 min. *NTC* no-template control. (**E**) Nucleotide sequence alignments of GP6+ (positions 5879 to 5903 according to HPV16 sequence, Genbank accession number NC_001526.4) to 13 HR-HPV genotypes. HPV genotypes are specified by numbers on the left. Dots indicate the presence of nucleotides identical to the top sequence (GP6+). HPV Types with weak amplification by the primer pool are shown by box.

According to the guidelines for nucleic acid testing of HPV released by Chinese Food and Drug Administration (CFDA) in 2015, 13 types of HR-HPV (types 16, 18, 31, 33, 35, 39, 45, 51, 52, 56, 58, 59, 68) are required to be identified when developing a diagnostic assay. We therefore designed a pool that includes 13 pairs of RPA primers targeting the L1 conservative region of the HR-HPVs (Table S1). To ensure a rapid and sensitive RPA reaction, all primers were synthesized at the length between 30 and 36 oligonucleotides. The kinetic fluorescence curve of Cas12a reaction showed that only a few HPV types generated strong signal, indicating weak amplification for most HPV types with this primer pool (Fig. S1). This could be due to the production of primer dimers, which consumed the materials in the reaction, and whereby reducing the intensity of specific amplification. We hypothesized that shorter and degenerated primers may reduce the complexity of the primer pool, and subsequently increase the amplification. The commonly used PCR primer sets meet such qualifications, but it is unknown whether they are compatible with the RPA system. As a proof-of-concept assay, we first evaluated the SPF1A, SPF1B and SPF2D primer combination (Fig. 2A, Table S2) in which the inosine was included to reduce the mismatches to targets^16^. However, no signal was observed when genomic DNA from siHA cell that is positive for HPV16 was tested (Fig. S2). We next evaluated another commonly used primer set GP5/GP6 for 6 types of HR-HPV detection. Although this primer set was able to amplify HPV16, HPV18 and HPV31, it failed to detect HPV33, HPV35 and HPV39, suggesting it was not a perfect candidate for all 13 types of HR-HPV detection (Fig. S3).

**Figure 3:**
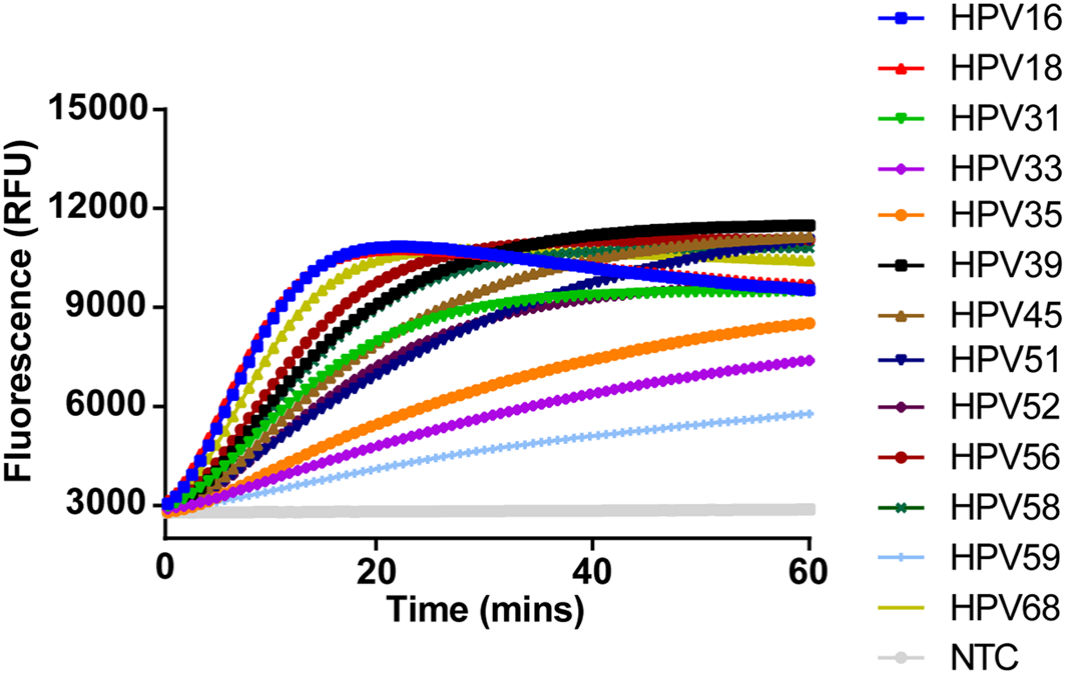
Evaluation of the enhanced PGMY/GP6+ primer pool. The PGMY/GP6+ primer pool was supplemented with additional four primers that have less mismatches to HPV31, 39, 51, 56, 59, 68. The new pool was tested using 10,000 copies of plasmid for each RPA reaction. ***NTC*** no-template control.

Then, we applied the PGMY/GP6+ primer pool to detect plasmids of HPV16 and HPV18 (Fig. 2A). As shown in Fig. 2B, this pool could detect both types with high sensitivity (100 copies for a single reaction). All crRNAs targeting the sequences flanked by PGMY/GP6+ were designed and validated with 5 μg of individual HPV plasmid (Fig. 2C). The primer pool was further tested for the rest of HR-HPVs using 10,000 copies for RPA amplification in a reaction. The result demonstrated that most types were efficiently amplified except HPV31, 39, 51, 56, 59, 68 (Fig. 2D). After aligned the GP6+ sequence to genomic sequence of these six HPV types, we found significant mismatches that may result in the failure of amplification (Fig. 2E). To expand the spectrum of this primer pool for other HR-HPVs, we supplemented additional four primers that have better matches to these six HPV types into the pool. With the new pool, all the 13 types of HR-HPVs were successfully detected when 10,000 copies of plasmid for each RPA reaction was used (Fig. 3).

**Figure 4:**
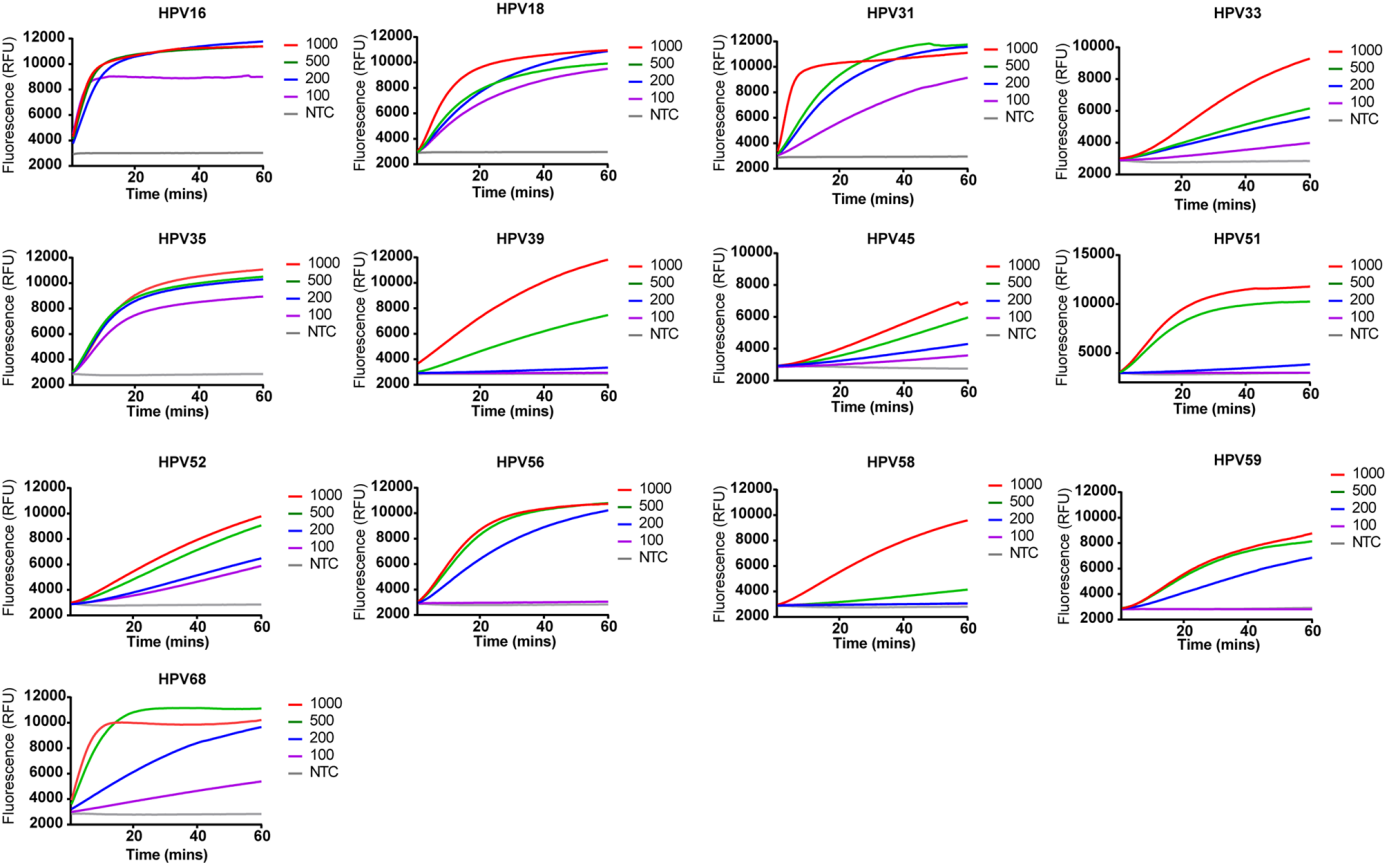
Serial dilutions of the indicated HPV plasmid for LoD determination. After RPA amplification at 37 °C for 20 min using 10,000 copies of plasmid for each reaction, 10 μL of the yield was transferred to 40 μL of the Cas12a mixture for cleavage assays. *NTC* no-template control.

The sensitivity of our assay was subsequently analyzed, and the results showed that it was able to detect all HPV types in a reaction containing 500 copies of plasmid (Fig. 4). A much higher sensitivity to HPV16, 18, 31, 35, 52, and 68 was achieved, where the limit of detection (LoD) was 100 copies for a given reaction (Fig. 4). Our assay seems not to be as sensitive as the reported ones in which the LoD of single molecule was observed^14,17^. The multiple RPA amplification could be the reason leading to the reduced sensitivity of the assay, which is consistent with a research by Zachary et al.^18^. The use of shorter primers could be another reason for the higher LoD in our assay, as shorter primers would be less efficient in amplifying the targets. The final optimized primer pool includes a total of ten primers (5 PGMY11 and 5 GP6+, Table S3), and the crRNA pool consists of 11 crRNAs, with one crRNA recognizing three HPV types (HPV18, 31, 33, Table S4). According to the instruction described in the protocol of Twist-Dx, the total amount of oligonucleotides in the reaction mixture should not exceed 1000 nM. However, the concentration of primers in current study (4000 nM in total) was far more than the standard, we therefore titrated the primers in reactions for detection of HPV16. The result showed that 4000 nM of primers in a reaction generated the strongest signal, indicating the highest amplification efficiency at this concentration (Fig. S4).

We next compared the sensitivity of the RPA-Cas12a-based method with the RPA-only method by detection of plasmid of HPV16. The RPA-only approach demonstrated an inferior sensitivity than RPA-Cas12a-based assay with LoD of 10^4^ copies in a reaction (Fig. S5). The performance of only Cas12a detection for HPV16 without RPA amplification was also assessed, and the results showed it could detect as low as 0.1 μg (equivalent to around 10^9^ copies) of HPV16 plasmids in a reaction. However, when the amount of plasmid decreased to 0.01 μg and 0.001 μg per reaction, there was almost no signal detected (Fig. S6). These results together illustrate the necessity of combining RPA amplification with Cas12a technology.

**Figure 5:**
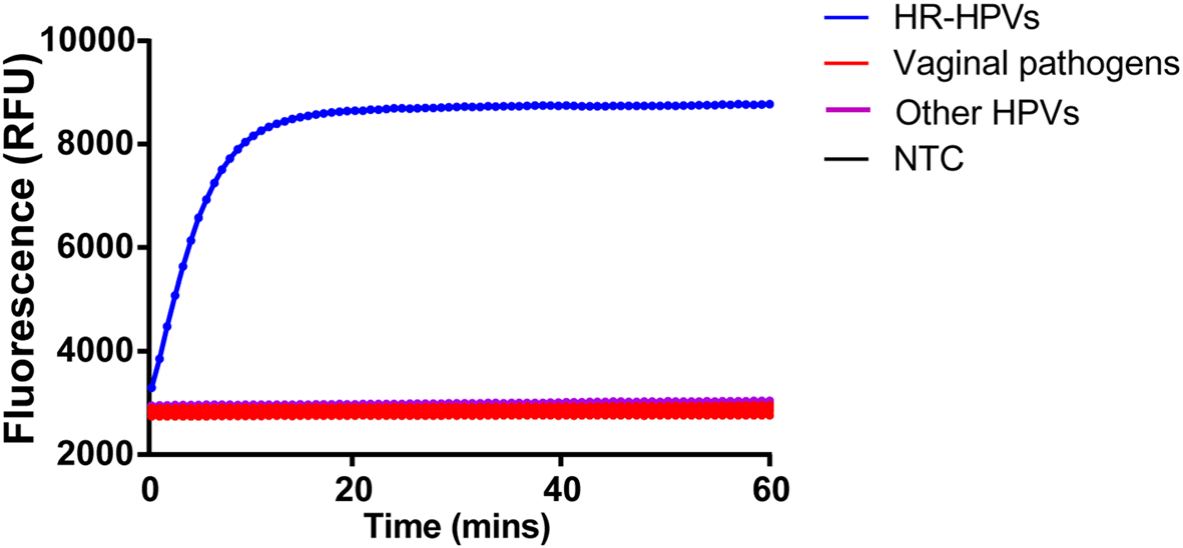
Specificity assessment of the RPA-CRISPR assay. Only DNA from the HR-HPVs produced signals, whereas plasmids from vaginal pathogens, other HPVs and the negative control did not produce any detected signals. The positive control was made by amplifying the HPV mixture where a total of 13 HR-HPVs were mixed at the concentration of 10,000 copies/μL for each type. *NTC* no-template control.

To interrogate any potential false positive, the specificity of our assay to 8 other HPV types (HPV6, 11, 26, 44, 54, 55, 61, 67) and 7 other pathogens commonly existed in vagina (chlamydia trachomatis, mycoplasma hominis, trichomonas vaginalis, treponema pallidum, streptococcus pyogenes, candida albicans, herpes simplex virus) was evaluated. As shown in Fig. 5, cross-reactivity was not observed with HR-HPVs and the tested pathogens, indicating high specificity of the assay.

**Figure 6:**
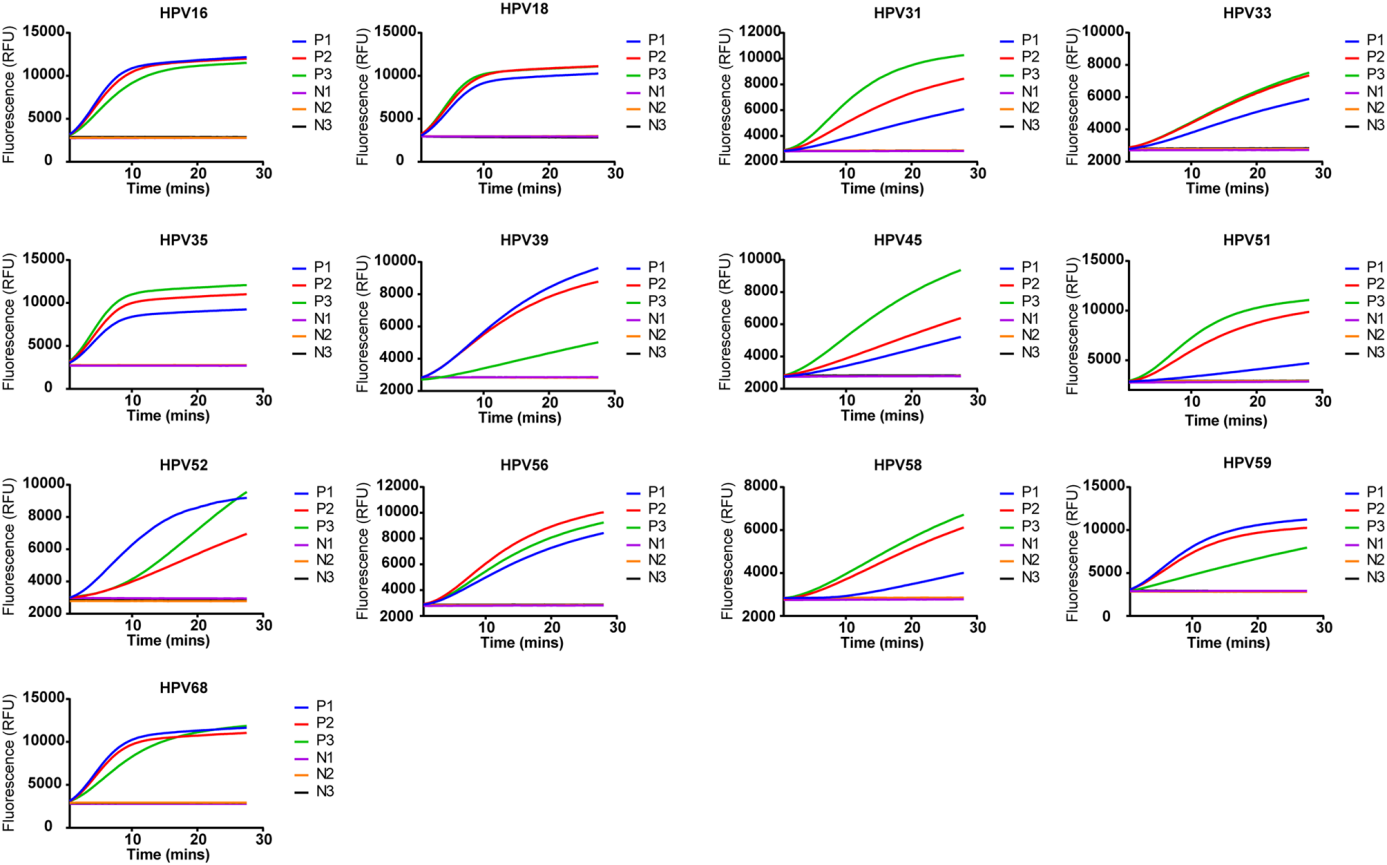
Clinical validation. Three positive and three negative samples identified by qPCR were used for testing for each type. The developed assay had 100% positive and negative agreements relative to the qPCR assay, for detection of the 13 types of HR-HPVs. *P* positive, *N* negative.

Finally, the clinical performance of the system was validated by analysis of materials from cervical scraps. All positive samples were only positive for one type of HR-HPV but either positive or negative for other HPV types determined by the HPV multiplex PCR testing. The negative samples were tested negative by the HPV multiplex PCR testing. All of the qPCR-positive samples were found positive in our assay, and all the negative samples were negative by our assay for detection of the 13 types of HR-HPV (Figs. 6, S7). Although the developed assay had 100% positive and negative agreements relative to the qPCR assay, the results should be carefully interpreted due to the limited number of specimens for each type. A larger cohort would be helpful to better understand the assay’s sensitivity and specificity in future studies.

In summary, we report the development of an RPA-Cas12a-based non-genotyping method for detection of 13 types of HR-HPV, which could serve as a useful tool for screening of cervical cancer. This method could also aid the diagnosis of other HPV-associated cancers, such as head and neck tumors. In contrast to the overall decrease in incidence of head and neck cancers in the United States, the incidence of HPV-associated oropharyngeal cancer appears to be increasing, highlighting the importance of this etiologic association^19^. An U.S. study found the incidence of oropharyngeal cancers (most likely to be HPV-associated) increased by 1.3% for base of tongue cancers and 0.6% for tonsillar cancers each year between 1973 and 2004^19^. The PCR approach remains the main method to identify the HPV infection for these diseases and limited availability of PCR test may contribute to the increasing rate of these diseases^20^. Since the proposed system is far more convenient than the PCR approach, the use of our system may help to revert this trend.

HPV DNA testing has been shown to offer better performance in the detection of cervical cancer than the commonly used Pap cytology testing^21^. However, current HPV tests, such as Cepheid Xpert HPV Assay and Roche’s cobas HPV test, require well-trained personnel, expensive instruments, and long reaction time (typically more than 2 h), which are not suitable for the applications at low-resource settings^22^. Besides the PCR-based HPV testing, several isothermal amplification based assays such as LAMP- and RPA-mediated HPV tests have also been developed^3,23–26^. Nonetheless, all these methods can just detect one type of HPV in a reaction, which greatly dampens their applications for HPV detection as more than 10 types of HPV are known to be closely associated with tumors^19,27^. Chen et al. combined the RPA amplification with the novel CRISPR system, and created an assay for detection of HPV16 and HPV18 (referred to as DETECTOR)^11^. Although DETECTOR has demonstrated high accuracy comparable to that of the PCR method, the same limitation was also faced with this system. Our proposed method was able to detect 13 types of HR-HPV simultaneously in a single reaction, which might be a very useful tool for diagnostic purposes.

The current study provides an easy approach for HR-HPV testing, however, this non-genotyping method may encounter substantial limitations since it is not able to genotype the most carcinogenic HPV types HPV16 and HPV18. To overcome this, additional reactions with primer sets of PGMY11-B and GP6+ for HPV16 and PGMY11-A and GP6+ for HPV18 could be performed (data not shown). Moreover, our method needs a tube-opening operation to transfer the RPA reaction for Cas12a cleavage, and this could generate aerosol and cause false-positive results. To avoid amplicon contamination, the tube opening should be strictly performed in a separate area. An alternative strategy was to add the Cas enzyme on the inner wall of the tube, and followed by a centrifugation step to start the Cas12a detection after RPA amplification^28^. Additionally, the current version of this assay does not include an internal control reaction, and thus false-negative may occur due to poor quality of the sample. A reaction for β-actin or GAPDH could be adopted to warrant the quality of the assay.

Our work also provides a novel concept to use RPA-based nucleic acid amplification. Previous study has realized triplex detections using the RPA technology even though the sensitivity was significantly reduced compared with the singleplex detection^18^. Nevertheless, when facing with more pathogen types, it is still a great challenge to apply the isothermal amplification-based methods for nucleic acid detection, particularly when using the RPA method. This might be due to the generation of primer dimers triggered by the second structure of primers as a result of too many primers in a single reaction. In present study, we created a primer pool and a crRNA pool that are capable of detecting 13 types of HR-HPVs in a reaction. The successful application of our method might be explained by the usage of the degenerated PCR primers that are shorter and less complex compared to the commonly used RPA primers. Thus, this novel methodology can be expanded for detection of multiple infections of other pathogens besides HPVs. To facilitate comprehensive applications, integration of the current method with microfluidic system would be necessary because in many cases there is a need for diagnostic technologies to be able to simultaneously test many targets. Xiong et al. reported a centrifugal microfluidic system, which could detect 32 targets in a single chip^29^. By combining with the microfluidic system, the capability of our method could be further expanded for the detection of multiple infections, such as detection of HR-HPVs together with other vaginal pathogens in a single assay.

## Materials and methods

### Nucleic acid preparation

The plasmid DNA containing full length HPV was extracted with a commercial kit (Tiangen, Wuxi, China) and the concentration was quantified using a spectrophotometer (Thermo Fisher, NJ, USA). The copy number of the plasmid was calculated by the following equation: DNA copy number = (M × 6.022 × 10^23^)/(n × 1 × 10^9^ × 650), where M is the amount of DNA in nanograms, n is the length of the plasmid in base pair, and the average weight of a base pair is assumed to be 650 Daltons.

### Primer pools

The conventional RPA primer pool contained 26 primers in equimolar quantity (2 μM for each, the sequences are shown in Table S1). The SPF primer pool is comprised of 3 primers (SPF1A, SPF1B, and SPF2D, shown in Table S2). As with the primer pool of PGMY/GP6+, a total of 6 primers were mixed at the concentration of 5 μM for each. The enhanced PGMY/GP6+ primer pool were made by supplementing additional 4 primers into the PGMY/GP6+ primer pool (Table S3).

### RPA reactions

RPA reactions were performed using a commercially available kit (Amp-Future, Weifang, China) according to the manufacturer’s protocol. Briefly, the reactions were performed at 37 °C for 20 min in a total volume of 12.5 µL comprising RPA enzymes, 1 µL of DNA input containing the indicated copy number of HPV and 100 ng of genomic DNA from C33A cells, 1× rehydration buffer, 14 mM magnesium acetate and 0.5 µL of each primer (10 μM) for the singleplex amplification, or 1 µL of the mixed primers (each at 5 μM) for the multiplex detection. As with the RPA-only detection, additional 120 nM probe specific for HPV16 was supplemented into the reactions.

### Cas12a detection reactions

The RPA product (10 µL) was transfered to 40 µL of the CRISPR-Cas12a reaction mixture containing 100 nM crRNA (Sangon Biotech, Shanghai, China), 50 nM Cas12a (NEB, Ipswich, UK) and 250 nM ssDNA reporter (Sangon Biotech, Shanghai, China). Then, the reactions (50 µL in a PCR tube) were incubated in a fluorescence plate reader (Molecular Devices, California, USA) or a Real Time PCR Detection System (Bio-Rad, Watford, UK) for up to 60 min at 37 °C with fluorescent signals collected every 30 s (ssDNA FQ substrates = λex: 485 nm; λem: 535 nm).

### crRNAs validation

A total of 11 crRNAs targeting the L1 region of HPVs were mixed in equimolar quantity each with the concentration of 50 nM, and this mixture was supplemented with 200 nM of the Cas12a enzyme. The sequences of all crRNAs were shown in Table S4. The solution was tested using 5 μg of the HPV plasmid in a reaction containing 20 μL of the solution and 5 μL of the plasmid.

### Analytical specificity analysis

The specificity of the assay was evaluated by testing DNAs from other HPV types (HPV6, 11, 26, 44, 54, 55, 61, 67), and other common vaginal pathogens (chlamydia trachomatis, mycoplasma hominis, trichomonas vaginalis, treponema pallidum, streptococcus pyogenes, candida albicans, herpes simplex virus). For each reaction, 10,000 copies of the synthetic DNA were used except the HR-HPV group where 1.3 × 10^5^ copies of the mixed HR-HPV plasmids were used with each type at the amount of 10,000 copies (13 types in total).

### Specimen collection and DNA extraction for HPV detection

A conventional cytological scrape was taken with a cytobrush from women visiting the gynecological outpatient clinic of the Shenzhen Luohu People’s Hospital in China. The specimens were placed into tubes containing 3 mL of cell collection medium (Yaneng Bio, Shenzhen, China) and stored at − 20 °C until use. 1 mL of the resuspended sample was used for DNA extraction with a commercial kit (Yaneng Bio, Shenzhen, China). Written informed consent was obtained from all enrolled patients.

### Ethical statement

The research on diagnostics for HPVs using clinical samples was approved by the ethical committee of Shenzhen Luohu People’s Hospital. All research was performed in accordance with the relevant guidelines and regulations.

## Supplementary Information

Supplementary Information.

## References

[1] Bosch FX, Lorincz A, Muñoz N, Meijer C, Shah KV (2002). The causal relation between human papillomavirus and cervical cancer. J. Clin. Pathol..

[2] Ginsburg O (2017). The global burden of women’s cancers: A grand challenge in global health. Lancet.

[3] Ma B (2019). A simple and efficient method for potential point-of-care diagnosis of human papillomavirus genotypes: Combination of isothermal recombinase polymerase amplification with lateral flow dipstick and reverse dot blot. Anal. Bioanal. Chem..

[4] Urquiza M (2005). Two L1-peptides are excellent tools for serological detection of HPV-associated cervical carcinoma lesions. Biochem. Biophys. Res. Commun..

[5] Hildesheim A (1994). Persistence of type-specific human papillomavirus infection among cytologically normal women. J. Infect. Dis..

[6] Manos M (1989). The use of polymerase chain reaction amplification for the detection of genital human papillomavirus. Cancer Cells.

[7] Qu W (1997). PCR detection of human papillomavirus: Comparison between MY09/MY11 and GP5+/GP6+ primer systems. J. Clin. Microbiol..

[8] Jiang HL, Zhu HH, Zhou LF, Chen F, Chen Z (2006). Genotyping of human papillomavirus in cervical lesions by L1 consensus PCR and the Luminex xMAP system. J. Med. Microbiol..

[9] Lee HP (2013). Comparison of the clinical performance of restriction fragment mass polymorphism (RFMP) and Roche linear array HPV test assays for HPV detection and genotyping. J. Clin. Virol..

[10] Satoh T (2013). Rapid genotyping of carcinogenic human papillomavirus by loop-mediated isothermal amplification using a new automated DNA test (Clinichip HPV). J. Virol. Methods.

[11] Chen JS (2018). CRISPR-Cas12a target binding unleashes indiscriminate single-stranded DNase activity. Science.

[12] Barrangou R (2007). CRISPR provides acquired resistance against viruses in prokaryotes. Science.

[13] Fozouni P (2021). Amplification-free detection of SARS-CoV-2 with CRISPR-Cas13a and mobile phone microscopy. Cell.

[14] Xiong D (2020). Rapid detection of SARS-CoV-2 with CRISPR-Cas12a. PLoS Biol..

[15] Broughton JP (2020). CRISPR-Cas12-based detection of SARS-CoV-2. Nat. Biotechnol..

[16] Kleter B (1998). Novel short-fragment PCR assay for highly sensitive broad-spectrum detection of anogenital human papillomaviruses. Am. J. Pathol..

[17] Gootenberg JS (2018). Multiplexed and portable nucleic acid detection platform with Cas13, Cas12a, and Csm6. Science.

[18] Crannell Z (2016). Multiplexed recombinase polymerase amplification assay to detect intestinal protozoa. Anal. Chem..

[19] Marur S, D'Souza G, Westra WH, Forastiere AA (2010). HPV-associated head and neck cancer: A virus-related cancer epidemic. Lancet Oncol..

[20] Shaikh MH, McMillan NA, Johnson NW (2015). HPV-associated head and neck cancers in the Asia Pacific: A critical literature review & meta-analysis. Cancer Epidemiol..

[21] Shin HY (2019). Evaluation of satisfaction with three different cervical cancer screening modalities: Clinician-collected Pap test vs. HPV test by self-sampling vs. HPV test by urine sampling. J. Gynecol. Oncol..

[22] Castle PE (2015). Reliability of the Xpert HPV assay to detect high-risk human papillomavirus DNA in a colposcopy referral population. Am. J. Clin. Pathol..

[23] Rohatensky MG (2018). Assessing the performance of a loop mediated isothermal amplification (LAMP) assay for the detection and subtyping of high-risk suptypes of Human Papilloma Virus (HPV) for oropharyngeal squamous cell carcinoma (OPSCC) without DNA purification. BMC Cancer.

[24] Yin K (2019). Synergistically enhanced colorimetric molecular detection using smart cup: A case for instrument-free HPV-associated cancer screening. Theranostics.

[25] Landaverde L, Wong W, Hernandez G, Fan A, Klapperich C (2020). Method for the elucidation of LAMP products captured on lateral flow strips in a point of care test for HPV 16. Anal. Bioanal. Chem..

[26] Ma B (2017). Isothermal method of a recombinase polymerase amplification assay for the detection of most common high-risk human papillomavirus type 16 and type 18 DNA. Clin. Lab..

[27] Mahmoodi P (2019). Early detection of cervical cancer based on high-risk HPV DNA-based genosensors: A systematic review. BioFactors.

[28] Wang B (2019). Cas12aVDet: A CRISPR/Cas12a-based platform for rapid and visual nucleic acid detection. Anal. Chem..

[29] Xiong H (2020). Rapid differential diagnosis of seven human respiratory coronaviruses based on centrifugal microfluidic nucleic acid assay. Anal. Chem..

