# Supplementary material for: A simple and rapid diagnostic method for 13 types of high-risk human papillomavirus (HR-HPV) detection using CRISPR-Cas12a technology
Source: Sci Rep. 2021 Jun 17;11:12800. doi: 10.1038/s41598-021-92329-2 (PMC8211657; doi:10.1038/s41598-021-92329-2)
Supplement: Supplementary file 1 — Supplementary Information. [file 41598_2021_92329_MOESM1_ESM.docx]

**Supporting information**

**A simple and rapid diagnostic method for 13 types of high-risk human papillomavirus (HR-HPV) detection using CRISPR-Cas12a technology**

Jiaojiao Gong^1#^, Guanghui Zhang^2#^, Wangguo Wang^3^, Liping Liang^2^, Qianyun Li^4^, Menghao Liu^5^, Liang Xue^1^*, Guanghui Tang^1^*

^1^ Yaneng Biotech, Co., Ltd, Fosun Pharma, Shenzhen, China.

^2^ Clinical laboratory, Shenzhen Hengsheng hospital, Shenzhen, China.

^3^ Department of infectious diseases, the Second People's Hospital of Shangrao, Jiangxi Province, Shangrao, China.

^4^ Department of Neurology, Hwa Mei Hospital, University of Chinese Academy of Sciences, Ningbo, China.

^5^ Nanobiological Medicine Center, Key Lab of Fuel Cell Technology of Guangdong Province, School of Chemistry and Chemical Engineering, South China University of Technology, Guangzhou, China.

^#^ These authors contributed equally to this work.

* Corresponding Authors: * tangguanghui@yanengbio.com (GT);

* xuel0620@sina.com (LX)

**Figure S1.** **Detection of 13 types of HR-HPVs using conventional RPA primer pool**

**Figure S2.** **Evaluation of SPF primer pool**

**Figure S3.** **Evaluation of the GP5/GP6 primer pool**

**Figure S4. Primer titration for the detection of HPV16**

**Figure S5. Evaluation of the RPA-only method for HPV 16 detection**

**Figure S6. Evaluation of the Cas12a-only method**

**Figure S7. Statistics of the clinical validation**

**Table S1. Conventional RPA primer pool**

**Table S2. SPF primer pool**

**Table S3. Enhanced PGMY/GP6+ primer pool**

**Table S4. crRNA pool and reporter used in this study**

**Ta**ble S5. **Probes used for RPA-only detection**


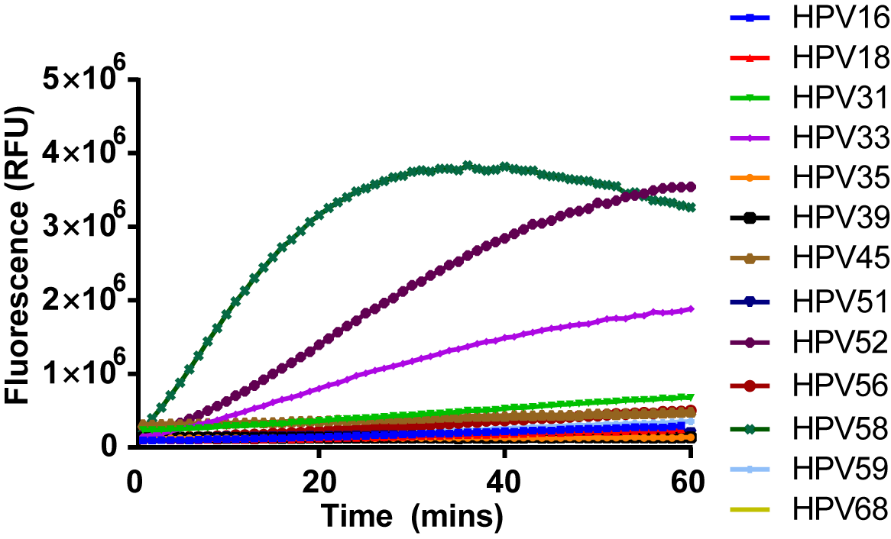


**Figure S1. Detection of 13 types of HR-HPVs using conventional RPA primer pool**

Representative plot of fluorescence intensity versus time for Cas12a detection of the HPVs. The RPA reaction was performed at 37°C for 20 min using the conventional RPA primer pool, and followed by the Cas12a cleavage assay at 37°C in a reaction containing 10 μL of the RPA amplicon and 40 μL of the Cas12a solution.


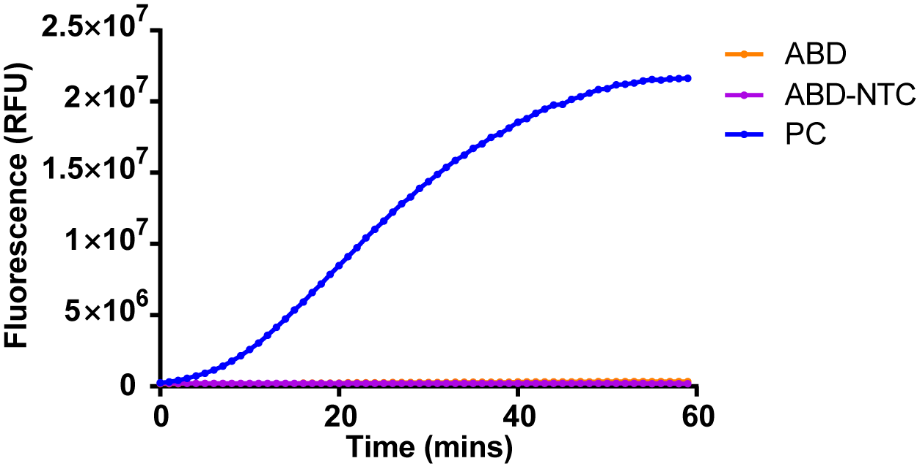


**Figure S2. Evaluation of SPF primer pool**

The SPF primer pool was tested using 10 ng of genomic DNA extracted from Siha cells which are positive for HPV16. The RPA reaction was conducted at 37°C for 20 min and followed by Cas12a detection at 37°C for up to 60 minutes. The positive Cas12a reaction was performed using 5 μg of the HPV16 plasmid. ABD: SPF1A, SPF1B and SPF2D; PC, positive control; NTC, no-template control.


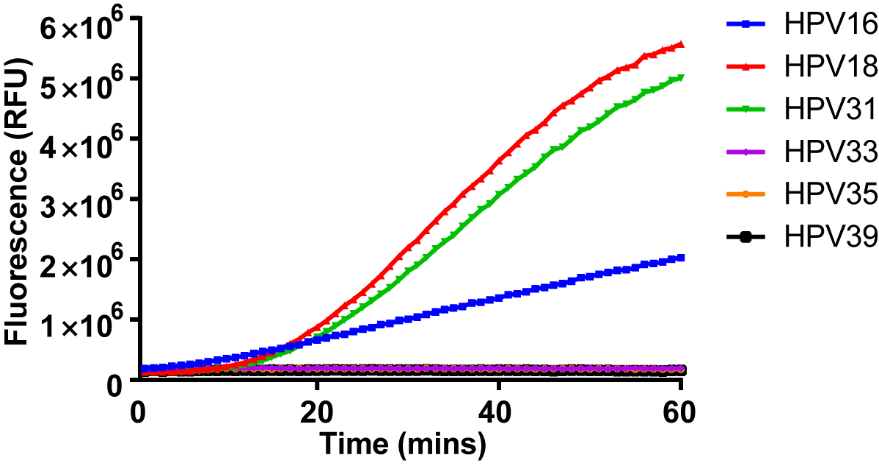


**Figure S3. Evaluation of the GP5/GP6 primer pool**

Six types of HR-HPVs were amplified using 10000 copies of plasmid for each RPA reaction. The RPA reaction was conducted at 37°C for 20 min and followed by Cas12a detection at 37°C for up to 60 minutes.


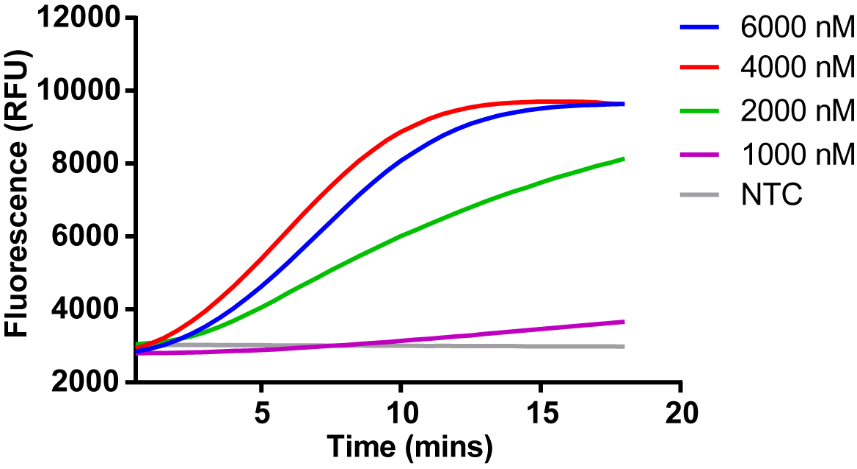


**Figure S4. Primer titration for the detection of HPV16**

The RPA was performed in a reaction with the indicated concentration of the enhanced PGMY/GP6+ primer set. Each reaction contained 10000 copies of plasmid for HPV16 detection. NTC, no-template control.


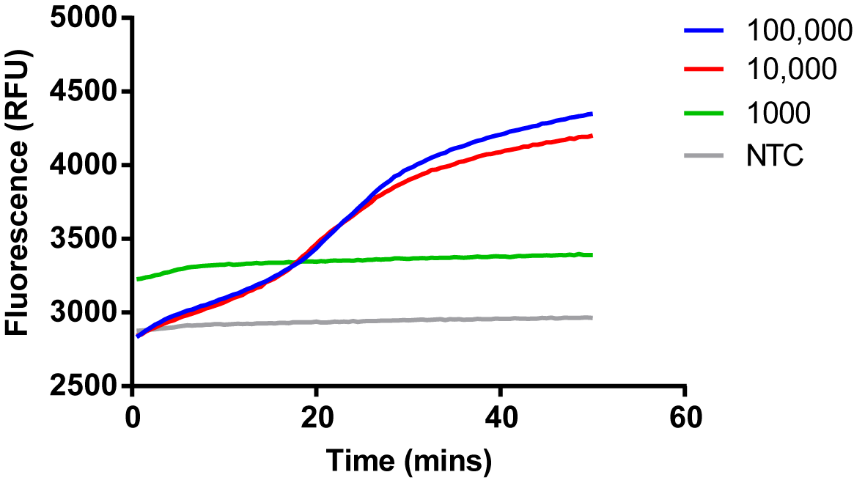


**Figure S5. Evaluation of the RPA-only method for HPV 16 detection**

The RPA-only detection was performed in a reaction with the indicated copy number of HPV16 plasmid. The primer pool of enhanced PGMY/GP6+ was used for the evaluation. NTC, no-template control.

**
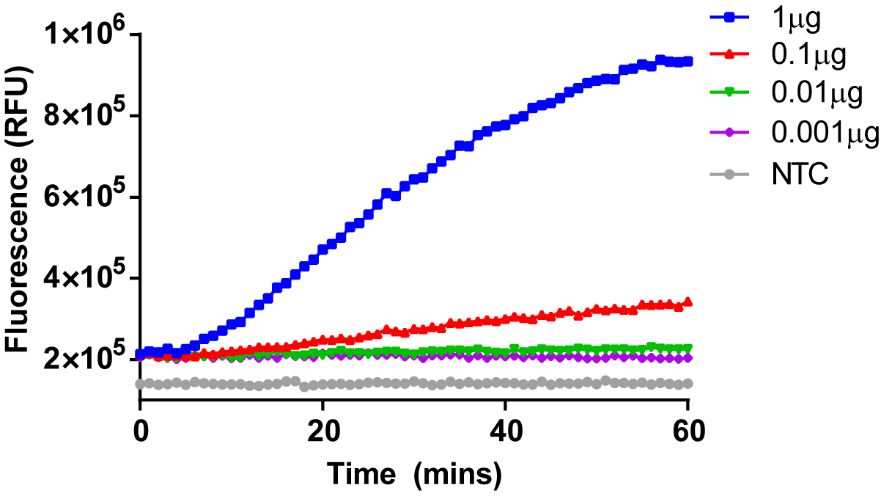
**

**Figure S6. Evaluation of the Cas12a-only method**

The fluorescence kinetics displaying the only Cas12a detection using the indicated amount of plasmid for HPV16. A reaction in a total volume of 25 μL containing 5 μL plasmid and 20 μL Cas12a solution was performed at 37°C for up to 60 minutes. NTC, no-template control.

**
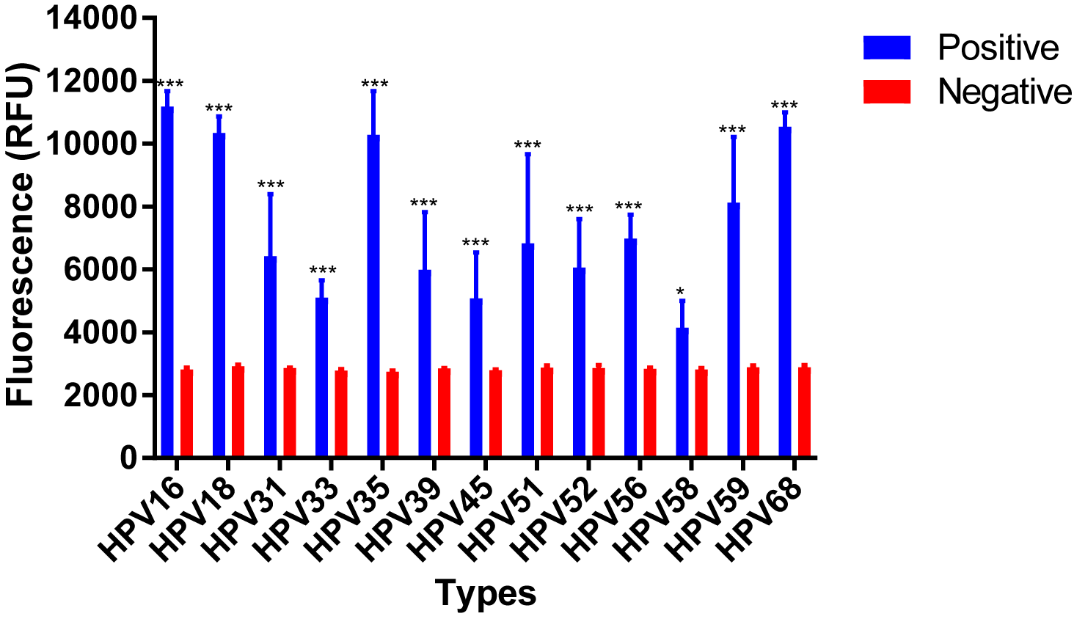
**

**Figure S7. Statistics of the clinical validation**

Fluorescent signal was obtained at 15 minutes for the Cas12a reaction. Three positive and three negative samples were used for each test. The data are presented as the means ± SD. Unpaired 2-tailed t test was used to analyze the difference between the positive and negative groups. *** P < 0.001, * P < 0.05

**Table S1. Conventional RPA primer pool**

| **Oligo name** | **Sequence(5'-3')** |
| --- | --- |
| RPApri-H16L1-F | GGTTACAACGAGCACAGGGCCACAATAATGGCA |
| RPApri-H16L1-R | CCCATGTCGTAGGTACTCCTTAAAGTTAGTATTTT |
| RPApri-H18L1-F | AATTATTTGTTACTGTGGTAGATACCACTCGCAG |
| RPApri-H18L1-R | CACAACTGAAAAATAAACTGCAAATCATATTCCTC |
| RPApri-H31L1-F | TTTCCTACACCTAGCGGCTCCATGGTTACT |
| RPApri-H31L1-R | GCAGCACACACAGACATATTGGTACTACGTGT |
| RPApri-H33L1-F | ACATTGCAGGCTAATAAAAGTGATGTTCCTAT |
| RPApri-H33L1-R | CCCTATTAAAAAAGTGTCTTACAAACATTTG |
| RPApri-H35L1-F | TTTCCTACTCCTAGTGGCTCTATGGTAACC |
| RPApri-H35L1-R | GCAGAACACACAGACATATTTGTACTACGGGT |
| RPApri-H39L1-F | AGGATGGTGATATGATTGATACTGGCTATGG |
| RPApri-H39L1-R | TGTTCCCTACGTAAACAGAAGAACATACTG |
| RPApri-H45L1-F | TAAACAGGCTGTTCCTAAGGTATCCGCATATCA |
| RPApri-H45L1-R | TCATCCAATTTATTATAAAATGGATGGCCACT |
| RPApri-H51L1-F | TGCTACTCCCAGTGGGTCTATGATAACATC |
| RPApri-H51L1-R | AAAAAGCTGATTGTTCCAGCAAATGCCATT |
| RPApri-H52L1-F | TCAGGAAATCCTGGGGATTGTCCTCCCCTACAAC |
| RPApri-H52L1-R | CATATGGCTCGCTAGCCATTTGCAAATAATCTG |
| RPApri-H56L1-F | CCTCCTAGTTCTGTATATGTTGCTACGCCTAGTG |
| RPApri-H56L1-R | ACTACAGTAACAAATAATTGATTACCCCAGCA |
| RPApri-H58L1-F | ACATTGCAGGCTAATAAAAGTGATGTGCCTAT |
| RPApri-H58L1-R | CCCTATTAAAAAAGTGTCTAACAAACATTTG |
| RPApri-H59L1-F | TAGACAGGATGTTCCTAAGGTGTCTGCATATCA |
| RPApri-H59L1-R | TCATCCAATTTATTATAGAATGGATGACCACT |
| RPApri-H66L1-F | CCTCCCAGTTCTGTATATGTTGCTACTCCTAGTG |
| RPApri-H66L1-R | ACAACAGTAACAAATACCTGATTACCCCAGCA |

**Table S2. SPF primer pool**

| **Oligo name** | **Sequence(5'-3')** |
| --- | --- |
| SPF1A | GCiCAGGGiCACAATAATGG |
| SPF1B | GCiCAGGGiCATAACAATGG |
| SPF2D | GTiGTATCiACTACAGTAACAAA |

i, inosine

**Table S3. Enhanced PGMY/GP6+ primer pool**

| **Oligo name** | **Sequence(5'-3')** |
| --- | --- |
| PGMY11-A | GCACAGGGACATAACAATGG |
| PGMY11-B | GCGCAGGGCCACAATAATGG |
| Modified PGMY11-C | GCACAAGGCCATAATAATGG |
| PGMY11-D | GCCCAGGGCCACAACAATGG |
| PGMY11-E | GCTCAGGGTTTAAACAATGG |
| GP6+ | GAAAAATAAACTGTAAATCATATTC |
| Modified PG6+ for H31, H39 | GAAATATAAATTGTAAATCAAACTC |
| Modified PG6+ for H51 | GAAAAATAAATTGCAATTCATACTC |
| Modified PG6+ for H56 | GAAAAACAAATTGTAATTCATATTC |
| Modified PG6+ for H59, H68 | GAAATATAAACTGCAAATCATATTC |

**Table S4. crRNA pool and reporter used in this study**

| **Oligo name** | **Sequence(5'-3')** |
| --- | --- |
| H16-L1-crRNA7 | UAAUUUCUACUAAGUGUAGAUuuacuguuguugauacu |
| H18/31/33-L1-crRNA | UAAUUUCUACUAAGUGUAGAUuuacugugguagauacc |
| H35-L1-crRNA2 | UAAUUUCUACUAAGUGUAGAUuuacuguaguugauaca |
| H39-L1-crRNA5 | UAAUUUCUACUAAGUGUAGAUaggaauauaccaggcacgugg |
| H45-L1-crRNA3 | UAAUUUCUACUAAGUGUAGAUagcaguauaguagacaugugg |
| H51-L1-crRNA3 | UAAUUUCUACUAAGUGUAGAUuuaccuguguugauacuaccag |
| H52-L1-crRNA4 | UAAUUUCUACUAAGUGUAGAUaggaauaccuucgucauggcg |
| H56-L1-crRNA5 | UAAUUUCUACUAAGUGUAGAUgugcaucauauuuacuuaacug |
| H58-L1-crRNA3 | UAAUUUCUACUAAGUGUAGAUaggaauauguacgucauguug |
| H59-L1-crRNA3 | UAAUUUCUACUAAGUGUAGAUacaguuguagauacuac |
| H68-L1-crRNA4 | UAAUUUCUACUAAGUGUAGAUaggaauauguuaggcauguuga |
| ssDNA FQ reporter | FAM-CCCCCC-BHQ1 |

**Ta**ble S5. **Probes used for RPA-only detection**

| **Oligo name** | **Sequence(5'-3')** | **Description** |
| --- | --- | --- |
| HPV16-Probe | GCAGTACAAATATGTCATTATGTGCTGCCAdT-FAM A H C dT-BHQ1ACTTCAGAAACTACA-C3 spacer | H, tetrahydrofuran |
